# Morphofunctional Assessment of Malnutrition and Sarcopenia Using Nutritional Ultrasonography in Patients Undergoing Maintenance Hemodialysis

**DOI:** 10.3390/medicina61061044

**Published:** 2025-06-05

**Authors:** José C. De La Flor, Estefanya García-Menéndez, Gregorio Romero-González, Celia Rodríguez Tudero, Elena Jiménez Mayor, Enrique Florit Mengual, Esperanza Moral Berrio, Beatriz Soria Morales, Michael Cieza Terrones, Secundino Cigarrán Guldris, Jesús Hernández Vaquero

**Affiliations:** 1 Department of Nephrology, Hospital Central de la Defensa Gómez Ulla, 28047 Madrid, Spain; jherva5@mde.es; 2 Department of Medicine and Medical Specialties, Faculty of Medicine, Alcala University, 28805 Madrid, Spain; beatriz.soria@edu.uah.es; 3 Health Sciences Doctoral Program, Faculty of Medicine, Alcala University, 28805 Madrid, Spain; 4 Department of Nephrology, Hospital Universitario Puerta de Hierro Majadahonda, 28222 Madrid, Spain; estefanialisset.garcia@salud.madrid.org; 5 Grupo REMAR-IGTP, Department of Nephrology, Hospital Germans Trias i Pujol, Instituto de Investigación Germans Trias i Pujol (IGTP), 08916 Badalona, Spain; garomerog.germanstrias@gencat.cat; 6 Department of Nephrology, Hospital Universitario de Salamanca, 37007 Salamanca, Spain; crodrigueztudero@usal.es; 7 PhD in Surgery Department, Faculty of Medicine, University of Salamanca, 37007 Salamanca, Spain; 8 Department of Nephrology, Hospital San Pedro de Alcántara, 10001 Cáceres, Spain; elena.jimenezm@salud-juntaex.es; 9 Department of Nephrology, Sistemes Renales Hemodialysis Clinic, 25198 Lleida, Spain; eflorit@sistemes-renales.com; 10 Department of Nephrology, Hospital General Universitario de Ciudad Real, 13005 Ciudad Real, Spain; emoral@sescam.jccm.es; 11 Department of Engineering, Faculty of Science and Engineering, Peruana Cayetano Heredia University, Lima 15002, Peru; michael.cieza@upch.pe; 12 Department of Nephrology, Hospital Ribera Polusa, 27004 Lugo, Spain; scigarran@riberacare.com

**Keywords:** morphofuntional assessment, malnutrition, sarcopenia, nutritional ultrasonography, hemodialysis

## Abstract

*Background and Objectives:* Malnutrition and sarcopenia are highly prevalent and clinically impactful conditions in patients undergoing maintenance hemodialysis (MHD), yet their early detection remains challenging. This study aimed to assess the diagnostic performance of nutritional ultrasonography (NUS) in the morphofunctional evaluation of malnutrition and sarcopenia, and to compare its utility with established tools such as bioimpedance analysis (BIA), biochemical markers, handgrip strength (HGS), and functional performance tests. *Materials and Methods:* A cross-sectional study was conducted in 74 stable MHD patients. Clinical, analytical, anthropometric, BIA, NUS, and functional parameters were collected, along with validated nutritional and frailty scales. NUS was used to assess the quadriceps rectus femoris (QRF) and preperitoneal visceral fat (PPVF), measuring *Y*-axis, *Y*-axis/height, cross-sectional muscle area rectus femoris (CS-MARF), muscle area rectus femoris index adjusted to height (MARFI_h_), and supramuscular fat (SMF). Sarcopenia was defined according to the 2019 European Working Group on Sarcopenia in Older People (EWGSOP) criteria. *Results:* The prevalence of risk, confirmed, and severe sarcopenia was 24.3%, 40.5%, and 20.3%, respectively. Severe-to-moderate protein-energy wasting (PEW) affected 44.6% of patients. Compared to non-sarcopenic individuals, sarcopenic patients had lower values of HGS, prealbumin, lean body mass, and phase angle. NUS-derived cut-off values for sarcopenia were *Y*-axis ≤ 8 mm, *Y*-axis/height ≤ 2.9 mm/m^2^, CS-MARF ≤ 2.4 cm^2^, and MARFIh ≤ 0.9 cm^2^/m^2^. The most discriminative NUS parameters were *Y*-axis and SMF (AUC 0.67), followed by *Y*-axis/height (AUC 0.65) and MARFI_h_ (AUC 0.63). NUS measurements correlated significantly with ASMI, phase angle, HGS, and SPPB scores. Conclusions: Nutritional ultrasonography is a feasible, reproducible, and clinically valuable tool for assessing muscle mass and quality in MHD patients. Its incorporation into routine practice may enhance early detection of malnutrition and sarcopenia, thereby facilitating timely, individualized nutritional interventions.

## 1. Introduction

Chronic kidney disease (CKD) is a major public health problem worldwide due to its high and increasing prevalence and the great impact it has on the morbidity and mortality of affected patients. In recent years, it has been estimated as one of the leading causes of death worldwide, with a significant rise over the last two decades [1,2,3]. As CKD progresses, patients require renal replacement therapies (RRT), such as peritoneal dialysis (PD) and hemodialysis (HD), the latter being one of the most used modalities. However, these procedures, although vital, are associated with various complications that adversely impact the patient’s health and quality of life and influence their nutritional profile, leading to differences in energy intake and glucose absorption. Among these complications, malnutrition stands out, affecting a considerable proportion of HD patients, being present in 20–70% of this population, depending on the study and the diagnostic criteria used [4]. According to the 2015 clinical guidelines of Clinical Nutrition of the European Society of Clinical Nutrition and Metabolism (ESPEN), malnutrition is defined as a state resulting from a lack of intake or uptake of nutrition that leads to altered body composition (decreased fat-free mass (FFM)) and body cell mass (BCM), leading to diminished physical and mental function and impaired clinical outcome from disease [5]. Malnutrition in CKD patients can include both undernutrition and overnutrition. Undernutrition, sarcopenia, and cachexia in CKD are complex, multifactorial nutritional disorders that involve both intrinsic and extrinsic factors [3]. These nutritional disorders can result from inanition, comorbidity, or muscle wasting due to immobility and ageing [6]. Undernutrition is associated with poor clinical outcomes, such as increased morbidity, longer hospital stays, readmissions, reduced quality of life, refractory anemia, frailty, and sarcopenia [7]. Whereas overnutrition, such as obesity, is a growing pathology worldwide not infrequent in HD patients, aggravating hypertension, diabetes, cardiovascular disease (CVD) and CKD [3].

In 2008, the International Society for Renal Nutrition and Metabolism (ISRNM) defined the term protein-energy wasting (PEW) as the pathological state characterized by the progressive and/or continuous decrease or depletion of both protein deposits and energy reserves, including loss of body fat and underlying catabolism [8]. On the other hand, The Global Leadership Initiative on Malnutrition (GLIM) introduced the GLIM criteria for early detection and treatment of malnutrition [9]. However, using these criteria to diagnose malnutrition is still under development and validation in patients with advanced chronic kidney disease (ACKD) on HD.

Sarcopenia is defined as a disease of skeletal muscle that results in loss of muscle mass and strength [10]. In January 2019, the European Working Group on Sarcopenia in Older People (EWGSOP) revised and updated the definition of sarcopenia. Key changes include the introduction of the concept of muscle quality, which is now given greater importance for diagnosing decreased muscle strength. Additionally, sarcopenia is no longer considered solely a geriatric syndrome but rather a disease of skeletal muscle. Furthermore, impairment of functional capacity is now considered a criterion of disease severity [11]. The prevalence of sarcopenia in hemodialysis patients varies from 4 to 64% [12]. The causes are diverse, resulting in an imbalance between muscle synthesis and catabolism. Risk factors such as malnutrition, medications that reduce appetite, and increased nutrient losses during dialysis contribute to sarcopenia [13].

Consequently, properly assessing malnutrition and sarcopenia in HD patients is essential for effective clinical management, as the early diagnosis and prompt treatment of these conditions can greatly enhance survival outcomes and quality of life. However, conducting nutritional assessments in this group of patients presents specific challenges due to their alterations in body composition. Traditionally, the assessment of malnutrition was based on anthropometric parameters such as body mass index (BMI), skinfold measurement, and arm and waist circumference. These parameters provide valuable information but still have limitations in dialysis patients (PD and HD) due to alterations in fluid distribution and body composition compartments [13]. The morphofunctional assessment of malnutrition is essential for comprehending the impact of inflammation, the accumulation of uremic toxins, and metabolic alterations. These factors significantly influence the body composition and nutritional status of patients with ACKD undergoing HD, enabling us to offer more effective care and support. The morphofunctional assessment integrates diagnostic or exploratory measures such as biochemical parameters, anthropometry, dynamometry, bioelectrical impedanciometry (BIA), nutritional anamnesis and physical exam, nutritional ultrasonography (NUS) of the muscle mass of the anterior quadriceps rectus femoris (QRF) and preperitoneal visceral fat (PPVF) and functional physical performance assessment [14,15,16].

This study aimed to evaluate the usefulness, in real clinical practice, of morphofunctional assessment with NUS for the diagnosis of malnutrition and sarcopenia in a cohort of patients on maintenance hemodialysis (MHD).

## 2. Materials and Methods

### 2.1. Study Design and Participants

This study is a descriptive observational analysis conducted at a single center, utilizing real-world data in a cross-sectional format. We included prevalent patients from the HD unit of the Nephrology Department of the Hospital Central de la Defensa Gómez Ulla between December 2024 and February 2025. The selected patients fulfilled the following inclusion criteria: patients undergoing MHD for over 90 days on RRT; aged 18 years or older; provided informed consent; and exhibited clinical stability, characterized by no hospital admissions in the past three months and the absence of active infectious or neoplastic conditions. The exclusion criteria encompassed patients with acute or chronic liver disease, pregnancy, acute or chronic infections, and active malignant diseases. Furthermore, patients who could not participate in any of the proposed tests—thus resulting in incomplete data on NUS, analytical assessments, anthropometric measurements, BIA, dynamometry, and functional capacity—were also excluded.

The main aim of our study was to assess the clinical utility of NUS (quality and muscle mass of the QRF, and PPVF) for diagnosing malnutrition and sarcopenia in patients undergoing MHD. The study sought to establish cut-off values for various ultrasound measurements of muscle using NUS in patients at risk of sarcopenia (rSA), as well as those with confirmed sarcopenia (cSA) and severe sarcopenia (sSA). A secondary objective was to determine the relationship between body fat and lean mass parameters measured by NUS, body composition techniques such as BIA, and muscle quality assessed by handgrip strength (HGS). Finally, the study also examined whether the differences in the muscle mass of the anterior QRF and PPVF measured by NUS could contribute to the diagnosis of sarcopenia.

This study received approval from the Drug Research Ethics Committee at the Hospital Central de la Defensa Gómez Ulla, under code number 50/24, on 22 November 2024, and was conducted in accordance with the principles of the Helsinki Declaration.

### 2.2. Data Collection

#### 2.2.1. Patient Characteristics and Analytic Variables

After signing informed consent, the patient’s clinical data were collected, such as age, sex, etiology of CKD, comorbidities, dialysis time, type of vascular access, HD modality, number and duration of HD sessions per week, type of dialyzer and dry weight. Laboratory data included blood count, total protein, albumin, C-reactive protein (CRP), lymphocytes, ferritin, transferrin saturation index (TSI), serum sodium (sNa^+^), serum chloride (sCl^−^), serum potassium (sK^+^), total serum calcium (sCa^++^), serum phosphorus (sP), serum intact parathyroid hormone (iPTH), serum urea (sU) and serum creatinine (sCr). Finally, efficacy data and HD parameters such as Kt/V, KT, infusion volume in post-dilution, blood flow rate (QB), arterial pressure flow (APF), and venous pressure flow (VPF) were collected.

#### 2.2.2. Anthropometric Variables

The anthropometric measurements obtained were weight in kilograms (kg), height in meters (m), and body mass index (BMI), calculated as weight/height × height (kg/m^2^). The arm circumference (AC) measurement in centimeters (cm) was obtained at the middle point between the acromion and radial head with a relaxed arm. The waist circumference (WC) was measured at the midpoint between the last costal border and the iliac crest, at the navel level, without pressure, by applying the tape measure horizontally (cm). Triceps and suprailiac skinfold thicknesses were measured following the procedures established by the International Society for the Advancement of Kinanthropometry (ISAK) protocol [17], using a Harpenden skinfold caliper (HSK-BI, British Indicators, Chichester, UK). Anthropometric data were collected by the same operator to eliminate inter-observer variation.

#### 2.2.3. Bioelectrical Impedanciometry Variables

The body composition and fluid status were analysed using a direct segmental multifrequency bioelectrical impedance device with an eight-point tactile electrode system, which took 30 impedance measurements using six frequencies (1, 5, 50, 250, 500, and 1000 kHz) across five segments (right arm, left arm, trunk, right leg, and left leg) (InBody S10, InBody Japan Inc., Tokyo, Japan). Measurement of body fluid balance included total body water (TBW), extracellular water (ECW), intracellular water (ICW), and the ratio of ECW to TBW (ECW/TBW). These were calculated using the formula provided in the software based on the measured height, weight, and impedance at 50 kHz [18]. The measurement of body composition included body fat mass (BFM), FFM, lean body mass (LBM), BCM, skeletal muscle mass (SMM), bone mineral mass, resistance (R), capacitance (X), and phase angle (PhA) at 50 kHz [19]. From the TBW values obtained via BIA, we estimated the appendicular skeletal muscle (ASM) using Lin’s formula [20]. We divided this result by the height (m^2^) to calculate the ASM index (ASMI). BIA measurements were taken 30 min after the end of the midweek hemodialysis session, with the patient in the supine position. According to the InBody S10 user manual, the body composition and body fluid parameters were calculated using a physiological calculation model: the body volume model for ICW, ECW, and TBW, and the body composition model for LBM and BFM parameters [19].

#### 2.2.4. Muscle Strength Variables

Muscle strength was evaluated by dynamometry, with the CAMRY dynamometer model EH101 (Zhongshan Camry Electronic Co., Ltd., Zhongshan, China) calculating the greatest of three HGS measurements. The measurement was performed with the patient seated, with their dominant or non-fistula hand positioned at right angles to the body. We considered decreased muscle strength in the palmar grip to be <27 kg in males and <16 kg in females, according to the 2019 EWGSOP2 consensus recommendations [11]. If muscle strength falls below these cut-off points, the individual is classified as being at risk of, or having probable, sarcopenia. Moreover, we also measured the 30 s Chair Stand Test (30s-CST), which serves as an indicator of leg muscle strength (quadriceps muscle group). This test involves the patient sitting in the center of a chair, with their hands crossed at shoulder level on the opposite wrist, feet flat on the floor, back straight, and arms close to their chest. They then rise to a full-standing position and sit back down. This is repeated for 30 s, with the number of times the patient can stand up and sit down counted [21].

#### 2.2.5. Functional Physical Performance Variable

The Short Physical Performance Battery (SPPB) was used to measure functional performance. The SPPB is a composite test that evaluates three simple exercises (balance test, 4 m gait speed test and five times rising from the chair test) performed in a specific order to avoid fatigue [22]. The maximum score is 12 points, with ≤8 points indicating poor physical performance [23].

#### 2.2.6. Nutritional Ultrasonography Variables

The ultrasound measurements were conducted using Digital Color Doppler Ultrasound equipment (Mindray Z60, Madrid, Spain), following the protocol previously published by García-Almeida et al. [16]. The NUS of the QRF and PPVF were performed with the patient in the supine decubitus position and the head of the bed at an inclination of 0 °. The measurements were carried out in B mode using a multifrequency linear probe (6–12 MHz) (Mindray Z60, Madrid, Spain).

**Figure 1:**
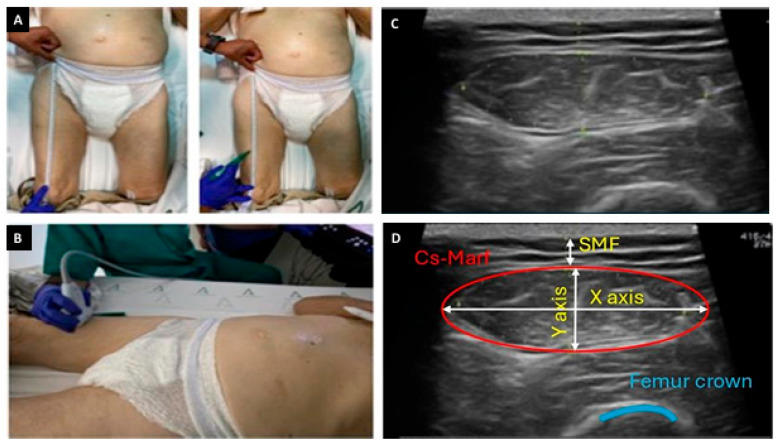
Systematic nutritional ultrasound study of the anterior quadriceps rectus femoris (QRF) muscle. The patient was in the supine decubitus position with the headrest at 0° with both legs extended and relaxed. An imaginary line was drawn between the anterosuperior iliac crest and the upper edge of the patella (**A**), and the transducer was placed in a transverse position to the axis of the leg in the lower third to measure the cross-sectional area of the anterior QRF (**B**). The QRF muscle and its ultrasonographic measurements (**C**,**D**). CS-MARF: cross-sectional muscle area of the rectus femoris; SMF: supramuscular fat.

First, the assessment of the anterior QRF was performed on the patient’s dominant leg. An imaginary line was drawn between the anterosuperior iliac crest and the superior border of the patella. The measurements were taken without compression, with the muscle relaxed, at the level of the lower third of this imaginary line, and with the transducer perpendicular to the longitudinal axis of the anterior QRF (Figure 1). The variables measured to assess muscle mass were anteroposterior muscle thickness (*Y*-axis in mm), adjusted for height (*Y*-axis/h in mm/m^2^) and patient’s body surface area (*Y*-axis/BSA in mm/m^2^); transversal muscle thickness (*X*-axis in mm); supramuscular fat (SMF in mm); and cross-section muscle area of the rectus femoris (CS-MARF in cm^2^). The CS-MARF was standardized by height [MARF (cm^2^)/(height × height) (m^2^)], which was named the muscle area rectus femoris index (MARFI_h_ in cm^2^/m^2^), which was then adjusted to BSA (MARF_BSA_ in cm^2^/m^2^). The Du Bois and Du Bois formula was employed to calculate the BSA [24]. The X-Y index, relating transversal and anteroposterior muscle thickness, was used to evaluate muscle quality (*X*-axis/*Y*-axis in mm). These parameters have been proposed in previous studies as relevant markers of muscle mass and body composition in patients with CKD and other clinical populations [16,25]. In our study, all ultrasound parameters were treated as continuous variables, since no universally accepted diagnostic cut-off values exist for the dialysis population.

**Figure 2:**
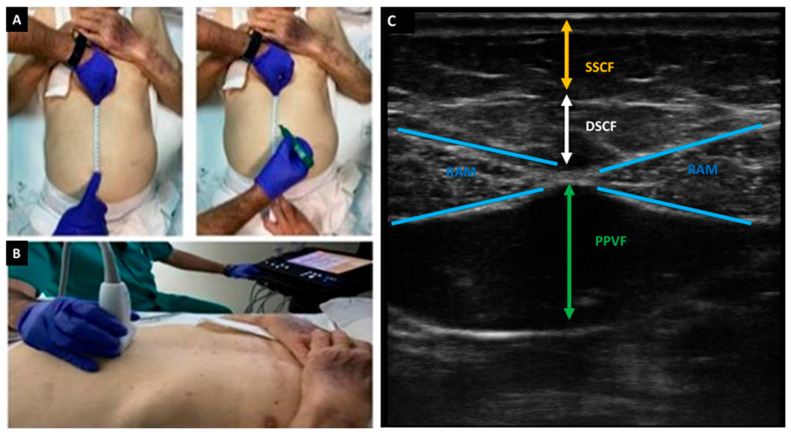
Systematic study (**A**,**B**) and ultrasound anatomy of preperitoneal visceral fat (PPVF) (**C**). SSCF: superficial subcutaneous fat; DSCF: deep subcutaneous fat; RAM: rectus abdominis muscle.

Secondly, for the measurement of PPVF, an imaginary line was drawn between the xiphoid appendix and the umbilicus. At the midpoint of this line, an image of the total transverse skin fat [superficial subcutaneous fat (SSCF in cm) plus deep subcutaneous fat (DSCF in cm)] and PPVF were obtained, with the transducer perpendicular to the longitudinal axis (Figure 2).

Due to the operator-dependent nature of ultrasonography, we acknowledge that intra- and inter-observer variability are important considerations when evaluating the reproducibility and clinical applicability of NUS in dialysis care, but when it is performed by a trained operator under consistent conditions, intra-observer variability is acceptable for clinical use. To minimize variability, all ultrasound examinations were performed by the same trained operator, using a standardized protocol as previously discussed, and always on the same day of the week (intermediate day for conventional HD 3 days a week, and the last day of the week for incremental hemodialysis (iHD)) to ensure consistent conditions. For each assessment, three measurements were taken and the mean value was used in the analysis to reduce random measurement error and enhance reliability.

#### 2.2.7. Malnutrition and Frailty Diagnosis

Malnutrition risk was assessed using the Malnutrition Screening Tool (MST), a scale of 0 to 5 points. Scores ≥ 2 indicate malnutrition risk, while 0 to 1 point indicates no risk [26]. This test evaluates appetite and involuntary weight loss, although it is not validated explicitly in HD patients. However, its speed and simplicity make it a useful tool for identifying malnutrition risk, enabling early nutritional interventions. Another screening tool employed was the 7-point Subjective Global Assessment (7p-SGA) scale, classified into three grades: very mild risk to well-nourished (6 or 7 in most categories), mild to moderate malnutrition (3, 4, or 5 ratings in most categories), and severe malnutrition (1 or 2 ratings in most categories) [27].

In addition, malnutrition was diagnosed using PEW-ISRNM-2014, as well as the malnutrition inflammation scale (MIS) in our HD patients. The PEW-ISRNM-2014 was determined based on the presence of one or more of the following: serum albumin ≤ 3.8 g/dL, BMI ≤ 23 kg/m^2^, sCr/BSA ≤ 3.8 mg/dL per m^2^ and nPCR ≤ 0.8/kg/per day [28]. Patients who did not meet any criteria received a score of 4 (normal nutritional status). Those meeting one criterion (score 3 = mild malnutrition), two criteria (score 2 = moderate malnutrition), or more than three criteria (score 1 or 0 = severe malnutrition) were categorized accordingly. Nevertheless, when the MIS scale was used, the normal range was ≤ 3 points; mild, 3–5 points; moderate, 6–8 points; and severe, ≥ 8 points [29].

On the other hand, frailty was evaluated using the FRAIL scale, which assesses five questions matching the acronym: fatigue, resistance, ambulation, illness, and loss of weight [30]. Patients were considered frail with a score ≥ 3 points, at risk of frailty with 1–2 points, and no frailty with 0.

#### 2.2.8. Sarcopenia Diagnosis

We used the Strength, Assistance in walking, Rise from a chair, Climb stairs and Falls (SARC-F) questionnaire as a screening tool to detect sarcopenia following the 2019 EWGSOP2 consensus recommendations [11]. The maximum score is 8 points, and the rSA is defined by the presence of an SARC-F score ≥ 4.

The three components required by EWGSOP2—low muscle strength (HGS < 16 kg in women and <27 kg in men) (see Section 2.2.4), low muscle mass (ASMI < 5.5 kg/m^2^ in women and <7 kg/m^2^ in men) (Section 2.2.3), and physical performance (Section 2.2.5) were all assessed in this study to confirm the diagnosis of sarcopenia [11]. According to the new EWGSOP2 consensus, when low muscle strength (criterion 1), rSA is detected. The diagnosis is confirmed if criterion 2 (low muscle mass) is met (cSA). sSA is considered if physical function or performance is also impaired (criterion 3) (SPPB ≤ 8 points).

### 2.3. Statistical Analysis

Continuous variables are presented as means with standard deviations or medians with interquartile ranges, depending on their distribution. Categorical variables are shown as frequencies or percentages. Comparisons between groups were performed using Student’s *t*-test, Mann–Whitney U test, ANOVA or chi-square test, based on the nature of the variables. Correlation analysis for quantitative variables was conducted using the Pearson correlation test. Receiver operating characteristic (ROC) curves were applied to NUS measures to estimate the ability of ultrasound to discriminate patients with sarcopenia, using the area under the curve (AUC) and its 95% confidence interval as an indicator. Cut-off points were determined based on a balance between sensitivity and specificity, as reflected by the Youden index. A *p*-value < 0.05 was considered statistically significant. The analyses were performed using the statistical package Stata v. 16.0. (Stata Statistical Software: Version 16, 2019. College Station, TX, USA: StataCorp LLC).

## 3. Results

### Baseline Patient Characteristics

Of the 84 prevalent patients in our HD unit, 74 were included for morphofunctional assessment with NUS, as per the selection criteria. The mean age was 73.1 ± 15.5 years, and the median time on dialysis was 31 (IQR 12–52) months. The majority, 87.8%, came from our ACKD clinic, while 2.7% and 9.5% were from PD and non-functioning renal transplants, respectively. Prevalent comorbidities included 50% with diabetes, 31.1% with a history of chronic obstructive pulmonary disease (COPD), 50% with ischemic heart disease, 98.7% with hyperparathyroidism secondary to CKD, and 13.7% with previous oncological processes that were not active at the time of the study. The causes of ESRD included: glomerular disease (*n* = 12; 16.2%), chronic interstitial nephritis (*n* = 6; 8.1%), polycystic kidney disease (*n* = 4; 5.4%), vascular (*n* = 6; 8.1%), diabetic kidney disease (*n* = 25; 33.8%), other causes (*n* = 7; 9.5%) and unknown etiology (*n* = 14; 18.9%). Most patients, 82.4%, had sessions 3 times per week (mean 642.4 min), while 17.6% were on iHD with one (*n* = 6, 8.1%) or two (*n* = 7, 9.5%) sessions per week. The majority received post-dilution hemodiafiltration online (95.9%), followed by conventional HD (2.7%) and expanded HD (1.4%).

Intradialytic anticoagulation was administered using low molecular weight heparin and unfractionated heparin (*n* = 10; 13.5%). Vascular access was via an arteriovenous fistula (41.9%) or a permanent catheter (58.1%). The mean KT and Kt/V urea were 52 (SD 6.4) liters and 1.6 (SD 0.2) respectively. The membranes used were high-flux asymmetric triacetate (41.9%), high-flux helixone (25.7%), high-flux polynephron (24.3%), high-flux polyamix (6.8%), and medium cut-off (1.4%). The mean weekly dose of subcutaneous epoetin alfa and intravenous paricalcitol was 5500 (IQR 3000–12,000) IU and 1.9 (SD 2.8) mcg, respectively. 17.6% (*n* = 13) of patients were on treatment with oral cinacalcet: 10.8% (*n* = 8) at a dose of 30 mg/day and 6.8% (*n* = 5) at a dose of 60 mg/day. 82.4% (*n* = 61) were receiving 25-hydroxycholecalciferol treatment and 14.9% (*n* = 11) were taking potassium binders. The remaining baseline characteristics, anthropometric data, analytical results, dynamometry findings, BIA and NUS values, functional capacity studies, malnutrition and sarcopenia risk scores, for the entire sample and by gender, are presented in Table 1A,B.

Most study variables showed no statistically significant differences between men and women, except for triceps skinfold values among anthropometric parameters. Regarding HD parameters, men had higher dry weight (*p* = 0.047), while women had longer HD vintage (*p* = 0.03). Compared to men, women showed significantly lower values for mean HGS, as well as most BIA parameters except for the ECW/TBW ratio, BFM, BCM, TBW/FFM, visceral fat area, and PhA. Concerning NUS measurements, women had lower values with statistically significant differences in *Y*-axis/height, *Y*-axis/BSA, CS-MARF, MARFI_h_, and MARFI_BSA_. However, in visceral fat NUS, women had higher transverse measurements of DSCF and SSCF but without statistical significance. PPVF was similar between the sexes. There were no statistical differences for biochemical parameters except for cholesterol, non-HDL-cholesterol and LDL-cholesterol, which were higher in women (Table 1B). Additionally, a positive correlation between ASMI and HGS was better in men (r = 0.44; *p* = 0.001) than in women (r = 0.37; *p* = 0.09) (Table 1B).

According to the malnutrition risk scales—7p-SGA (mild–moderate–severely malnourished), MIS ≥ 8 points, and MST ≥ 2 points—50% (*n* = 37), 40.5% (*n* = 30), and 21.6% (*n* = 16) of the patients were classified as malnourished, respectively. Additionally, 39.2% (*n* = 29) and 20.3% (*n* = 15) of the patients were classified as frail and at risk of frailty, respectively. The prevalence of mild PEW (score 0–3) and severe–moderate PEW (score 0–2) were 89.2% and 44.6%, and according to the SARC-F score ≥ 4 points, the prevalence of rSA was 35.1% (*n* = 26). The prevalence of rSA, cSA, and sSA, according to 2019 EWGSOP2 criteria, were 24.3%, 40.5%, and 20.3%, respectively. Statistically significant differences (*p* = 0.01) were found in the distribution of sarcopenia categories (non-SA, rSA, and cSA) between sexes, with higher percentages of non-SA and cSA among men compared to women, whereas rSA was more common among women (Table 1B).

We compared the variables of the morphofunctional assessment in relation to the diagnosis of non-SA, rSA, and cSA patients. We observed significant differences in anthropometric (age, sex, weight, BMI, triceps, and suprailiac skinfold), muscle strength (HGS), and functional performance (SPPB) parameters. cSA patients had lower nPCR (*p* = 0.001) and KT (*p* = 0.02) values compared to those with rSA and non-SA. Regarding analytical parameters, cSA patients displayed lower levels of sCr (*p* = 0.007), triglycerides (*p* = 0.01), and prealbumin (*p* = 0.51) compared to rSA and non-SA patients. Analysis of body composition and fluid status measured by BIA revealed lower values of TBW, BFM, FFM, LBM, BCM, skeletal muscle mass, visceral fat mass, ASMI using Lin’s formula, and phase angle in cSA patients compared to non-SA and rSA patients, with statistically significant differences. The muscle mass and PPVF’s NUS measures of *Y*-axis (mm) (7.8 (2.3) vs. 8.8 (2) vs. 9.6 (2.8); *p* = 0.01), *Y*-axis/height (mm/m^2^) (2.9 (1) vs. 3.5 (0.7) vs. 3.4 (1.3); *p* = 0.03), CS-MARF (cm^2^) (2.4 (0.8) vs. 2.6 (0.6) vs. 2.9 (1); *p* = 0.03), SMF (6 (2.2) vs. 7.3 (1.8) vs. 7.5 (2.1); *p* = 0.02), transverse PPVF (cm) (0.5 (0.3) vs. 0.6 (0.2) vs. 0.7 (0.3); *p* = 0.05), SSCF (cm) (0.7 (0.3) vs. 0.8 (0.2) vs. 0.9 (0.3); *p* = 0.04), and DSCF (cm) (1.1 (0.5) vs. 1.4 (0.4) vs. 1.1 (0.6); *p* = 0.05) were significantly lower in cSA patients compared with rSA and non-SA, respectively (Table 2A,B).

*Y*-axis (mm), *Y*-axis/BSA (mm/m^2^), MARFI_h_ (cm^2^/m^2^), and SMF (mm) significantly discriminate SA from non-SA patients (all *p* < 0.05). However, *Y*-axis/BSA (mm/m^2^), MARFI_BSA_ (cm^2^/m^2^), transverse PPVF (cm), and DSCF (cm) failed to discriminate cSA from non-SA patients (all *p* > 0.05) (Table 3).

**Figure 3:**
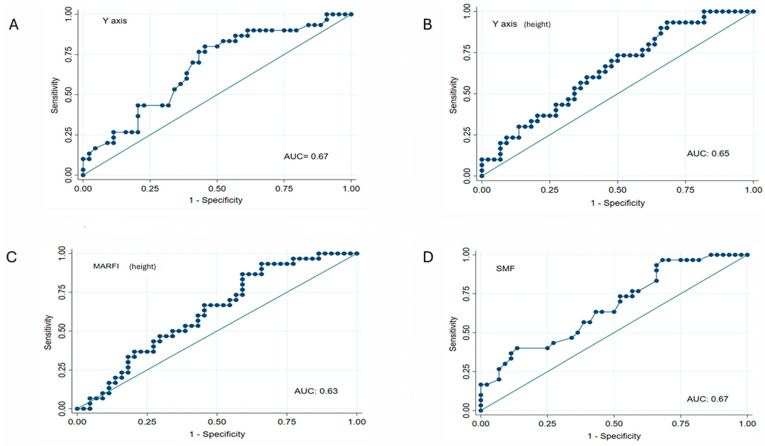
Predictive ability of nutritional ultrasound measurements of the cross-sectional muscle area of the rectus femoris (*Y*-axis (**A**); *Y*-axis (height) (**B**); MARFI (height) (**C**); and SMF (**D**)) for the diagnosis of sarcopenia according to EWGSOP2 consensus. *Y*-axis (height): *Y*-axis adjusted to height; MARFI: muscle area of the rectus femoris index adjusted to height; SMF: supramuscular fat; AUC: area under curve.

The parameters with the best discriminative power of NUS measurements of the rectus femoris muscle cross-sectional area for the diagnosis of sarcopenia according to the EWGSOP2 consensus were *Y*-axis (AUC 0.67; 95% CI: 0.54–0.79), *Y*-axis/height (AUC 0.65; 95% CI: 0.52–0.77), MARFI_h_ (AUC 0.63; 95% CI: 0.50–0.75), and SMF (AUC 0.67; 95% CI: 0.54–0.79) (Figure 3).

**Figure 4:**
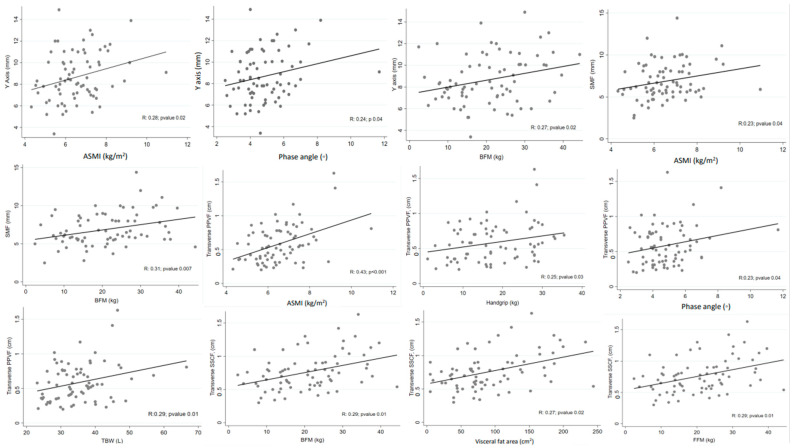
Correlation analysis of the values obtained by NUS (rectus femoris muscle mass and preperitoneal fat) with BIA parameters. ASMI: appendicular skeletal mass index; BFM: body fat mass; SMF: supramuscular fat; PPVF: preperitoneal visceral fat; TBW: total body water; FFM: fat-free mass.

Correlation analysis of the values obtained by muscle mass and PPVF’s NUS with BIA parameters showed that the *Y*-axis had a positive correlation with ASMI, phase angle, and BFM, while LBM or TBW did not correlate. Similarly, SMF showed an adequate correlation with BFM and ASMI. Transverse PPVF positively correlated with ASMI, handgrip, phase angle, and TBW. Transverse SSCF correlated with BFM, FFM, and visceral fat area (Figure 4).

## 4. Discussion

To our knowledge, this is the first real-world study that evaluates the usefulness of morphofunctional assessment with NUS for diagnosing malnutrition and sarcopenia in patients on MHD. It is also the first study to identify associations between NUS measurements and sarcopenia diagnosis according to the 2019 EWGSOP2 consensus criteria. The morphofunctional assessment approach considers not only body composition but also functional status, incorporating parameters such as BIA variables, along with biochemical indicators, HGS, and functional testing. Therefore, adopting a comprehensive and multimodal strategy for evaluating malnutrition and sarcopenia in this population is essential. Early diagnosis and treatment are critical for improving clinical outcomes. Thus, in this context, NUS has emerged as a valuable tool to assess both muscle mass and PPVF, as well as overall nutritional status. Our study demonstrated that in a cohort of patients on MHD, ultrasonographic parameters of QRF muscle mass, including *Y*-axis, *Y*-axis/height, CS-MARF, MARFI_h_, and SMF, were feasible for detecting sarcopenia, particularly in cases of confirmed sarcopenia [11]. Additionally, the *Y*-axis of QRF muscle mass positively correlated with BIA parameters such as ASMI, BFM, and PhA. The NUS of the PPVF also correlated with ASMI, HGS, PhA, and TBW. Finally, we estimated ultrasound cut-off values of the QRF as references for diagnosing sarcopenia in MHD patients, mainly *Y*-axis ≤ 8 mm, *Y*-axis/height ≤ 2.9 mm/m^2^, CS-MARF ≤ 2.4 cm^2^, and MARFI_h_ ≤ 0.9 cm^2^/m^2^. Although the discriminative power of some NUS parameters was modest (AUC 0.63–0.67), they may still hold clinical value as first-line screening tools. Given its non-invasive nature, low cost, and bedside applicability. NUS may help identify patients at risk of sarcopenia who could benefit from further assessment using functional and compositional criteria [14].

Malnutrition and sarcopenia are common complications affecting patients with ESKD undergoing RRT (HD and PD) [31]. Early identification is therefore essential to enable timely and effective therapeutic interventions. The morphofunctional assessment of malnutrition was designed with this goal in mind. It includes a set of diagnostic tools to evaluate both body composition and functional status, offering a detailed quantitative and qualitative analysis with prognostic and diagnostic relevance in states of both overnutrition and undernutrition. However, the use of NUS as a strictly morphological component within this framework is not yet fully established in routine clinical practice for patients on MHD. The 2020 Kidney Disease Outcomes Quality Initiative (KDOQI) guidelines on nutrition in CKD recommend a comprehensive nutritional assessment within 90 days of starting dialysis, annually or when clinically indicated, to identify those at risk of PEW or any other malnutrition alterations [32]. The guidelines support the use of the 7-point Subjective Global Assessment (7p-SGA) and the MIS for malnutrition screening, with 1B and 2C levels of evidence, respectively. Although the PEW score may be helpful in identifying subgroups of patients with poor nutritional status and high mortality rates, its applicability in the early detection of PEW remains uncertain [32]. In our study, we estimated the prevalence of malnutrition and PEW in a cohort of MHD patients based on three criteria: the 7p-SGA, MIS ≥ 8 points, and severe PEW (score 0–2). The corresponding prevalence rates were 50%, 30%, and 44.6%, respectively. These results indicate a high burden of nutritional impairment in our center, consistent with previous studies that reported similar prevalence rates, ranging from 28% to 80% [33,34,35,36].

Malnutrition prevalence varies according to the nutritional assessment tool employed, as well as across studies, and is also influenced by geographical and cultural factors that may explain such differences [37]. Severe malnutrition is a pathological condition in which undernourishment and hypercatabolism converge. In our cohort, the prevalence of severe PEW was lower than that reported by Gracia-Iguacel et al., who found a prevalence of 63% [38]. However, our findings are consistent with the meta-analysis by Carrero et al., which reported a prevalence ranging from 28% to 54% [39]. Notwithstanding, the prevalence in those centers was obtained using the SGA and MIS to assess PEW in CKD patients requiring dialysis. In contrast, a recent cross-sectional study of dialysis patients in Catalonia, Spain, using a new and practical online tool (Nutrendial), estimated a 23.3% prevalence of PEW (26% HD, 10.2% PD) [40].

Sarcopenia is strongly associated with increased rates of disability, fragility, and mortality [41]. While it is primarily age-related, it can also result from metabolic or endocrine disorders and chronic conditions such as heart failure, CKD, diabetes, and liver cirrhosis. In patients undergoing MHD, sarcopenia is multifactorial, driven by dialysis-specific factors such as chronic inflammation, oxidative stress, metabolic acidosis, and hormonal dysregulation. The prevalence of sarcopenia in MHD patients varies widely, ranging from 1.5% to 68% [12,42]. This variability is likely due to differences in study design, diagnostic criteria, patient demographics (e.g., sex, ethnicity), and type of RRT (HD or PD) [43]. In our cohort, based on the 2019 EWGSOP2 criteria [11], 40.5% of MHD patients met the criteria for cSA, and 20.3% for sSA. Our findings are consistent with the results from a recent meta-analysis by Duarte et al. [44]. This study reviewed 140 studies, and the 15 articles that used the EWGSOP2 criteria reported a prevalence of sSA in dialysis patients of 26.2% (95% CI: 16.6–37.1), slightly higher than the prevalence observed in our cohort. However, our findings are higher than those reported by Wathanavasin et al. [45], whose meta-analysis found a sarcopenia prevalence of 21.4% (18.0–31.4%) in 8 studies (*n* = 948 patients in dialysis) using the 2019 EWGSOP2 criteria, though they did not differentiate between cSA and sSA. Furthermore, they only mention that physical performance was evaluated in nearly half of all studies. Regarding the estimated prevalence of sarcopenia by RRT modality and CKD stage, HD patients have a higher frequency of sarcopenia than those with better renal function [46], while PD patients exhibit a significantly lower prevalence of sarcopenia than MHD patients [47]. A recent observational study by Garcia-Menendez et al. [48] found, in 38 PD patients, a sarcopenia prevalence of 36.1% according to 2019 EWGSOP2, slightly lower than our cohort (40.5%). The meta-analysis by Wathanavasin et al. [45] reported a sarcopenia prevalence in PD of 17.5%, similar to the 23.4% found by Shu et al. [49]. These findings suggest that certain features of PD, such as younger age, fewer comorbidities, a better overall health status, and greater preservation of residual renal function, may help maintain muscle mass and function more effectively than HD [43]. This trend was also observed in our cohort: patients on HDi with preserved residual renal function had a better nutritional profile than those on conventional HD. This was reflected in significantly lower rates of malnutrition based on the following criteria: MIS (7.7% vs. 47.5%, *p* = 0.008), SGA (30.1% vs. 54.1%, *p* = 0.13), severe PEW (23.1% vs. 49.2%, *p* = 0.09), and 2019 EWGSOP2-defined sarcopenia (5% vs. 25%, *p* = 0.06).

Also, our findings align with previous research. In a representative multicenter cohort, Slee et al. reported that over 40% of HD patients exhibit muscle wasting and sarcopenia, based on diagnostic criteria incorporating HGS, gait speed, and BIA-derived skeletal muscle indices [50]. These results underscore the clinical relevance and diagnostic complexity of sarcopenia in this population. Similarly, in our cohort, over 40% of patients met the criteria for confirmed sarcopenia, with parallel declines in muscle strength, PhA, and ASMI. Additionally, the methodological framework described by Janssen et al. for estimating skeletal muscle mass via BIA provided a validated reference for correlating our ultrasonographic measurements [51]. The observed associations between rectus femoris ultrasound parameters (e.g., *Y*-axis) and BIA-derived metrics (ASMI, PhA) support the potential of nutritional ultrasonography as a complementary, non-invasive tool for morphofunctional assessment in MHD.

Our analysis indicates a higher risk of sarcopenia among men compared to women. This finding aligns with previous studies suggesting that male sex may specifically influence sarcopenia-related traits, such as low HGS [44]. For instance, a retrospective study by Hung et al. of 325 PD patients found a higher prevalence of sarcopenia in men (25.1–75.6%) than women (2.2–31.3%), as determined by DEXA composition analysis [52]. This aligns with the hypothesis proposed by Duarte et al. that men in a uremic state may be more susceptible to loss of appetite, inflammation, and subsequent musculoskeletal imbalances [44].

Diagnosing sarcopenia requires assessing muscle mass, strength, and physical performance. According to GLIM, DEXA, and BIA are the two most widely recommended methods for measuring skeletal muscle mass [9]. DEXA, considered the gold standard for measuring body composition, measures SMM using low-dose X-ray beams to distinguish between bone, fat, and lean tissue. However, its high cost limits its availability in routine clinical settings. In contrast, BIA is a non-invasive, fast, and inexpensive method that estimates fat tissue index, lean tissue index, and hydration status based on electrical conductivity. BIA can also estimate ASM mass using formulas, as demonstrated by Lin et al., who validated an equation to calculate the ASMI in HD patients, using DEXA as a reference [20]. BIA is a well-established tool for assessing malnutrition and sarcopenia in dialysis patients [53]. The 2020 KDOQI guidelines on nutrition in CKD recommend the use of MF-BIA for assessing body composition when available [32]. However, it is important to note that the measurements obtained through BIA are estimates derived from electrical parameters, such as resistance, reactance, and PhA, combined with personal data like weight, height, age, and sex to calculate body mass and volume. This methodology has some limitations, including the need for specific consumables and contraindications, such as in patients with pacemakers. Moreover, in patients with ESRD receiving RRT, hydration status overestimates SMM measurements, which is a significant limitation in settings where fluid balance is variable. Therefore, BIA should ideally be performed at least 30 min after the end of a haemodialysis session to allow for the redistribution of body fluids, or with an empty abdomen in PD patients [32]. Similarly, NUS measurements may also be affected by tissue hydration, particularly in edematous patients, although this effect is less pronounced than with BIA. In our study, all assessments were performed post-dialysis to minimize the impact of fluid-related variability.

The patients diagnosed with sarcopenia in our study exhibited significantly reduced BIA measurements, characterized by low parameters in TBW, BFM, FFM, LBM, BCM, SMM, visceral fat area, ASMI and PhA. These results are consistent with the study by Cioffi I. et al., which reported that FFM and PhA are closely linked to functional status and nutritional condition, although they may vary with hydration [54]. PhA, obtained through BIA, reflects cell membrane integrity, BCM, and the ECW/ICW ratio. A PhA < 4.6° is considered the most accurate marker of PEW by ISRNM criteria, with a sensitivity and specificity of 86.4% and 73.6%, respectively [55]. Previous studies have demonstrated the association between PhA, mortality, and malnutrition in patients undergoing MHD. Furthermore, a prospective 2-year follow-up study in 250 MHD patients revealed that for every 1° increase in PhA, the risk of hospitalization and cardiovascular events was reduced by 21% and 30%, respectively [55]. In our cohort, sarcopenic patients had a mean phase angle of 4.3 (SD = 1.5).

NUS has emerged as a promising tool for assessing sarcopenia in recent years. NUS has emerged as a promising tool for assessing sarcopenia in recent years. Compared to BIA, NUS offers several advantages in clinical settings. It is portable, less expensive, and provides direct evaluation of muscle morphology independent of hydration status [32,56,57]. While BIA remains widely used for assessing body composition, NUS adds complementary structural information that may improve diagnostic precision in dialysis patients [58,59]. Its use in HD units is feasible with standard ultrasound devices and basic training for operators [32]. These findings support growing evidence that NUS is a valuable technique for morphofunctional evaluation of sarcopenia in CKD patients. Moreover, combining NUS with BIA may improve diagnostic accuracy and reduce misclassification due to fluid overload. The quadriceps femoris, especially the anterior QRF, is the most commonly assessed muscle. However, other muscles, such as the biceps and gastrocnemius, can also be examined using specific scanning protocols [56]. This involves scanning various muscle areas in transverse and longitudinal sections, allowing the assessment of muscle thickness, volume, area, fascicle length, echogenicity, and angle of pennation [57].

Thus, NUS of the anterior QRF provides direct information not only on muscle mass but also on muscle quality. It allows the detection of atrophy, fatty infiltration (myosteatosis), and fibrosis. NUS has been shown to correlate well with DEXA, MRI, and CT in estimating muscle mass in elderly patients [58]. Recent studies have also demonstrated that NUS is an effective method for measuring muscle mass using CS-MARF, which correlates with FFM measured by BIA, HGS by dynamometry, and physical performance [59]. However, there is limited evidence on the validity and reliability of NUS in populations with ACKD on RRT, which would allow the evaluation of abnormality patterns and cut-off points to enable early detection of muscle wasting in this population.

Several anterior QRF muscle mass parameters measured by NUS, such as CS-MARF, MARFI, and muscle thickness (*Y*-axis and *X*-axis), have been studied to differentiate between sarcopenia and PEW, primarily in patients with DRM and nutritional risk [40,60,61], and in a few studies in patients with ACKD receiving RRT [48,62]. Lopez-Gomez et al. conducted a cross-sectional study in 144 DRM patients according to GLIM criteria [60]. They performed a morphofunctional nutrition assessment using anthropometric variables, HGS by dynamometry, BIA, and NUS of the QRF. The prevalence of sarcopenia in their sample was 33.3%. They observed that sarcopenic patients had lower values of MARFI_h_ compared to the non-SA patients [1.09 (0.39) cm^2^/m^2^ vs. 1.27 (0.45) cm^2^/m^2^; *p* = 0.02], as well as lower values for the *Y*-axis [8.8 (2.7) mm vs. 11.9 (6.0) mm; *p* < 0.01] and *X*-axis/*Y*-axis ratio [1.52 (0.61) vs. 1.30 (0.53); *p* < 0.01]. These findings are consistent with our results, which showed very similar data for the *X*-axis/*Y*-axis ratio but lower values for the *Y*-axis [cSA: 7.8 (2.3) mm vs. non-SA: 9.6 (2.1) mm; *p* = 0.01] and MARFI_h_ [cSA: 0.9 (0.3) cm^2^/m^2^ vs. non-SA: 1 (0.4) cm^2^/m^2^; *p* = NS]. In the recent DRECO study (Disease-Related caloric-protein malnutrition EChOgraphy), de Luis Roman et al. evaluated the usefulness of NUS of the QRF in detecting sarcopenia in hospitalized patients at risk of malnutrition and defined cut-off values for NUS measures [61]. According to MUST, patients at high risk of malnutrition underwent a dynamometer HGS strength testing, BIA, a Timed Up and Go test (TUG), and NUS (QRF). According to the 2019 EWGSOP2 criteria, the study evaluated 991 out of 1000 total subjects, identifying 9.7% with confirmed sarcopenia and 3.9% with severe sarcopenia. The CS-MARF, *X*-axis, *Y*-axis, and *X*-axis/*Y*-axis ratio cut-off values for each sarcopenia category, without differentiating by sex, were as follows: for rSA, 3.37 cm^2^, 37.37 mm, 9.59 mm, and 5.19; for probably-SA, 3.37 cm^2^, 33.55 mm, 9.59 mm, and 4.63; for cSA, 3.66 cm^2^, 38.3 mm, 9.66 mm, and 4.19; and for sSA, 3.41 cm^2^, 38.3 mm, 8.77 mm, and 4.19.

The cut-off values for confirmed sarcopenia in our study were higher than the NUS parameters of the quadriceps femoris, including CS-MARF (2.4 cm^2^), *Y*-axis (7.8 mm), *X*-axis (30.2 mm), and *X*-axis/*Y*-axis ratio (1.5). These differences highlight the importance of defining condition-specific cut-off points. In patients on HD, lower thresholds may be more appropriate for diagnosing sarcopenia.

In the HD population, a recent cross-sectional study by Nagy et al. involving 41 Egyptian patients used NUS to measure CS-MARF. The prevalence of sarcopenia was 58.5%. Sarcopenic patients had significantly lower CS-MARF compared to those without sarcopenia, at 2.23 (1.36–4.5) cm^2^ vs. 3.49 (1.89–5.5) cm^2^, respectively (*p* = 0.001) [63]. These data are very similar to our findings. Furthermore, Matsuzawa et al. examined the validity of NUS (QRF) for assessing muscle mass and its clinical applicability as a diagnostic tool for sarcopenia in HD patients [64]. The study included 58 patients on conventional MHD. CS-MARF measured at the distal third of the femur showed a strong correlation with BIA-derived measurements and was independently associated with HGS (beta = 4.22; 95% CI = 2.23–6.20; *p*< 0.001), gait velocity (beta = 0.15; 95% CI = 0.05–0.26; *p* = 0.006), chair rise time (beta = 4.33; 95% CI = 7.34–1.31; *p* = 0.006), and SPPB score (beta = 1.81; 95% CI = 0.46–3.15; *p* = 0.010), even after adjusting for patient characteristics. The cut-off values for CS-MARF to identify HD patients with skeletal muscle loss were < 1.88 cm^2^ for men and < 1.43 cm^2^ for women, as determined by the Youden index. The NUS-based criteria (QRF) for low muscle mass yielded a sensitivity of 0.74, specificity of 0.94, positive predictive value of 0.96, negative predictive value of 0.63, and diagnostic accuracy of 0.80. Among those diagnosed with sarcopenia based on NUS (QRF), 96% also met the BIA-based diagnostic criteria. These cut-off values are substantially lower than those observed in our study, where all patients with cSA had CS-MARF values of 2.4 cm^2^.

Additionally, a multicenter study by Sahathevan et al. reported that a CS-MARF value below 5.21 cm^2^ (IQR: 4.10–6.21), measured at the mid-femoral point, was associated with an eightfold increased probability of diagnosing PEW syndrome in HD patients [62]. The cut-off values in this study were higher than ours, likely due to methodological differences, particularly in the anatomical site of measurement. While Sahathevan et al. [62], measured the CS-MARF at the mid femur, our protocol involved the distal one-third of the femur, which is more commonly used in the literature. This highlights the need for standardized NUS protocols to ensure homogenized samples and comparable cut-off values, enabling results to be extrapolated. Recently, a study by Garcia-Menendez et al. aimed to describe the utility of NUS of the QRF muscle for detecting and monitoring sarcopenia in PD [48]. The authors proposed that QRF thickness (*Y*-axis) is the most relevant parameter for estimating and monitoring muscle mass in sarcopenic PD patients. They identified a cut-off value of <8.5 mm (SD 2.4) for patients at risk of sarcopenia. The *Y*-axis was more discriminative than QRF cross-sectional area, as sarcopenic muscle tends to be more flaccid, preserving the *X*-axis but losing thickness. Our findings are similar. In MHD patients with cSA, *Y*-axis thickness was lower (<7.8 mm, SD: 2.3) compared to the values reported in the PD population.

In our study, muscle mass ultrasound parameters (SMF, MARFI_h_, *Y*-axis) showed a strong correlation with functional measures such as hand grip strength and SPPB, as well as with body composition assessed by BIA, compared to traditional biochemical markers. These findings confirm their usefulness as indicators of functional status and the severity of sarcopenia. Particularly noteworthy are their advantages over scales like 7p-SGA, MIS, and PEW, which, despite their widespread acceptance, have well-documented limitations regarding sensitivity, subjectivity and ability to capture muscle structural alterations [62]. In our study, the assessment of muscle mass by NUS using the *Y*-axis correlated with muscle mass measures determined by BIA, such as ASMI, PhA and BFM—findings very similar to those obtained in the study by Lopez-Gomez et al., with the difference that the MARFI_h_ was the NUS measure that correlated with those BIA parameters [60]. In the AnyVida trial, low values of phase angle measured by BIA and muscle mass measured by NUS were considered prognostic factors for mortality at 12-month follow-up in cancer patients [65]. It is worth noting that the measurement of both the SMF of the thigh and the transverse PPVF of the abdomen correlated adequately with parameters measured by BIA (ASMI, PhA, BFM, FFM and TBW), allowing us to assess not only muscle mass but also to have a reference of overall body fat. This association reinforces the value of NUS as an essential tool for morphofunctional assessment in HD patients. It suggests that its combination with analytical tests and functional measures, such as strength or physical performance, should be prioritized to enable a more accurate and personalized diagnostic assessment of nutritional status [62]. On the other hand, some studies such as Vogt et al. and Isoyama et al. in patients with ACKD on dialysis, have shown a greater association with muscle strength and mortality than with muscle mass [42,66]. In a systematic review and meta-analysis of ACKD patients with and without RRT, published by Ribeiro et al., the authors investigated the association between sarcopenia and its characteristics with mortality and hospitalization. Of the 4922 studies obtained from five electronic databases, including MEDLINE and Embase, 50 studies were included in the review (72,347 patients), with 38 of these also included in the meta-analysis (59,070 patients). The authors concluded that low muscle strength, low muscle mass, and low physical performance were associated with increased mortality in ACKD patients with or without RRT and that diagnosed sarcopenia also represented an increased mortality risk [67].

Indeed, another relevant aspect assessable by NUS of the QRF in HD patients is muscle quality, evaluated through echogenicity and the *X*-axis/*Y*-axis ratio. These measures help identify intramuscular fat (IMF), also known as myosteatosis. Traditionally, IMF has been assessed using imaging techniques such as CT, MRI, and ultrasound, typically targeting muscle groups in the legs, arms, and trunk. Recently, Avesani et al. [68] published a narrative review emphasizing IMF as a key marker of muscle quality IMF is associated with reduced contraction power and lower force generation per unit of muscle mass. This supports the hypothesis that loss of muscle strength is not solely due to reduced muscle mass, but also to qualitative changes, including fat infiltration.

In chronic kidney disease, IMF has been linked to decreased muscle strength, impaired muscle quality, metabolic abnormalities, cardiovascular disease, and increased mortality. Interventions aimed at reducing IMF in CKD are still emerging, but guided exercise may improve muscle quality in hemodialysis patients. One of the aims of this narrative review on IMF is to highlight the need for clinical trials assessing muscle in CKD patients to consider not only muscle quantity and morphology but also muscle quality. The study by Lopez-Gomez et al. also evaluated muscle quality by echogenicity and *X*-axis/*Y*-axis ratio, finding an inverse correlation with handgrip strength (r = −0.36; *p* < 0.01). In their multivariate analysis adjusted for age, the highest quartile of the *X*-axis/*Y*-axis ratio had a greater risk of death [OR: 4.54, 95% CI (1.11–18.47)] [60]. These findings are similar to those reported in studies of older adults by Bunout et al. [69] and patients with multiple sclerosis by Mañago M. et al. [70]. Interestingly, our study found no significant differences in muscle quality, measured by the *X*-axis/*Y*-axis ratio, between cSA and non-SA patients.

Regarding laboratory nutritional markers (albumin, transferrin, lymphocytes), the findings of our study align with recent literature that cautions about their limited sensitivity and specificity as isolated indicators of nutritional status [71]. Serum albumin, traditionally considered a marker of protein status, is significantly affected by non-nutritional factors such as chronic inflammation, fluid overload, and infections. These limitations reduce its utility as a specific indicator of malnutrition [72]. Transferrin, due to its shorter half-life, may offer a more dynamic reflection of protein status. However, its levels are also influenced by inflammatory states and iron deficiency. The use of lymphocyte count has been questioned due to its poor specificity and the influence of various immunological and pharmacological factors, particularly in immunocompromised patients like those under HD. Additionally, other biomarkers like prealbumin, C-reactive protein, and CRP/prealbumin ratio have been proposed as potential tools to differentiate between inflammation and malnutrition. However, their use is not standardized, and they do not substitute a direct structural assessment of muscle tissue. In this context, nutritional ultrasound provides a complementary and more precise approach, as it enables direct evaluation of muscle volume, structural quality (through echogenicity), and symmetry between limbs, aspects that no biochemical marker can provide [71]. This has been highlighted in the 2020 KDOQI clinical guidelines on nutrition in CKD [32], which state that no single marker or method alone can provide a complete and unequivocal assessment of nutritional status and recommend a combination of subjective and objective assessment methods. Correlations between NUS parameters and nutritional or inflammatory biomarkers, such as albumin, transferrin, and lymphocyte count, were examined and discussed. These markers showed limited association with structural ultrasound measures, highlighting the added clinical value of NUS in the morphofunctional assessment of dialysis patients [57,71].

Some authors even consider sarcopenia the physical substrate of frailty. Reduced muscle mass and strength contribute directly to frailty, a clinical condition defined by increased vulnerability resulting from the loss of physiological functions [73]. Frailty is highly prevalent among dialysis patients, significantly more so than in the general population and those with chronic kidney disease not requiring renal replacement therapy. It is generally accepted that the frequency of frailty in this setting is around 50% [74]. In our cohort, frailty assessed using the FRAIL scale was present in 39.2% of patients. Among those with sarcopenia, the prevalence rose to 60%. Frail individuals face an increased risk of falls, infections, hospitalizations, surgical complications, and mortality [43]. Kamijo et al. evaluated the association between sarcopenia and frailty in a cross-sectional and longitudinal study involving 119 patients on PD [75]. Frail patients exhibited lower ASMI, HGS, and usual walking speed. The presence of frailty in HD patients was linked to a poorer prognosis, which could be an additional factor to consider when deciding on initiating RRT or whether conservative management would be more appropriate for each individual patient [74].

The new approach to nutritional status, centered on morphofunctional assessment in MHD patients, evaluates changes in body composition and function, allowing a global assessment of the individual. Noteworthily, when used alone, each of these nutritional indicators can lead to inaccurate and delayed diagnoses. This is an accessible, safe, low-cost tool with a relatively rapid learning curve, facilitating implementation in clinical practice. It assesses muscle function and quality, providing morphological and metabolic measurements.

The use of NUS in nutritional assessment broadens diagnostic options and enables monitoring of muscle loss and gain in the QRF during therapeutic interventions. Current intervention strategies for sarcopenia in MHD patients focus on slowing muscle mass and strength loss while preserving the ability to perform daily activities through protein supplementation, physical exercise, and pharmacological treatment. The 2020 KDOQI clinical guidelines on nutrition in CKD recommend a protein intake of 1.0–1.2 g/kg/day for HD patients [32]. Several nutritional strategies have been implemented to improve protein intake in sarcopenic HD patients, including oral nutritional supplements (ONS), amino acids, leucine, whey protein, enteral, and parenteral nutrition [43,76]. A systematic review and meta-analysis by Liu et al. of 22 studies with 1185 dialysis patients found that the ONS group exhibited significantly increased serum albumin [1.26 g/L (95% CI, 0.50–2.02, *p* < 0.0001; I^2^ = 80.4%)], BMI [0.30 kg/m^2^ (95% CI, 0.09–0.52, *p* = 0.005; I^2^ = 41.4%)] and HGS [0.96 kg (95% CI, 0.07–1.84, *p* = 0.034; I^2^ = 41.4%)] from baseline to the end of intervention. The review concluded that the use of oral nutritional supplements can improve the nutritional status of dialysis patients, as reflected in increased serum albumin, BMI, and HGS, without significant effects on serum phosphorus, potassium, and C-reactive protein levels [77]. Matsuzawa et al. conducted a meta-analysis of four studies exploring the effects of oral nutritional supplements and parenteral nutrition on muscle mass, muscle strength, and physical function in patients undergoing MHD. The results of this meta-analysis showed that protein supplementation positively impacted physical performance, but did not influence muscle mass or strength [78]. Moreover, the Kidney Disease: Improving Global Outcomes-2024 Clinical Practice Guideline for the Evaluation and Management of CKD recommends physical activity for patients with CKD stages 1–5. Physical activity has been shown to improve physical performance, cardiorespiratory fitness, and quality of life [79]. In this context, combining oral energy and protein supplementation with supervised exercise offers a comprehensive strategy to counter sarcopenia in MHD patients. Several therapeutic interventions such as vitamin D, angiotensin receptor blockers, myostatin inhibitors, and anabolic steroids—have also been proposed. However, despite encouraging experimental evidence, these approaches remain underutilized in clinical practice [43].

The main limitations of our study were the small sample size and its cross-sectional, single-center design, which may restrict the generalizability of the results. Furthermore, this evaluation was conducted at a single time point without considering the evolution of the technique, which does not allow us to predict the risk of hospitalization or mortality in those patients diagnosed with sarcopenia. Another important limitation is the demographic data of our patients, which may influence the generalization of the results, given that the sample is limited to a specific population from a given area of the community of Madrid in Spain with particular sociodemographic characteristics. In addition, the different modalities of HD, both conventional and incremental, to which our patients are subjected, could affect the presentation and evaluation of sarcopenia, introducing variability in the findings. Therefore, the information from our study can only be applied to patients with malnutrition and sarcopenia undergoing these HD modalities and cannot be extended to peritoneal dialysis, given the study’s design. Further, our study did not include a comparison between NUS and gold standard methods for assessing muscle mass (e.g., DEXA, MRI). This limitation, inherent to the real-life nature of the study, may reduce the precision and reliability of our findings. Although all measurements were performed post-dialysis to minimize variability, we cannot entirely rule out the influence of residual fluid overload on NUS parameters. Another potential limitation is the operator dependence of NUS, as its accuracy and reproducibility are influenced by the experience of the examiner. However, using a standardized protocol by a single evaluator provides internal validity to the study. The main strength of this study is the incorporation of NUS within the planning of the morphofunctional assessment, which allows us to better understand the quality, mass, and muscle function of patients on MHD. Multicenter research is needed to increase statistical power and reduce variability between centers, with the goal of establishing more accurate reference values for diagnostic tests for sarcopenia in the kidney disease population, specifically in MHD patients.

## 5. Conclusions

Our study underscores the utility of NUS as an accurate, accessible, and complementary diagnostic tool for evaluating nutritional status and identifying sarcopenia in patients undergoing MHD. The findings demonstrate that ultrasonographic parameters of the anterior QRF muscle exhibit significant correlations with both functional performance and body composition metrics, thereby addressing key limitations inherent to conventional techniques such as BIA and standard biochemical markers.

Importantly, the capacity of ultrasound to detect structural muscle alterations—such as fatty infiltration and changes in echogenicity—offers a qualitative diagnostic advantage, enhancing its value beyond that of purely quantitative modalities. The consistency of our results with previous studies further validates the reliability of ultrasound as a cornerstone in morphofunctional assessment, particularly when combined with established functional tools like the SPPB and HGS tests.

In light of these findings, we advocate for the integration of NUS into the routine morphofunctional evaluation of MHD patients. As part of a multidimensional clinical framework, NUS enables a more comprehensive assessment that encompasses structural, functional, and biochemical dimensions. This approach holds significant potential to improve the early detection and management of malnutrition and sarcopenia in this high-risk population.

## Figures and Tables

**Table 1:** (**A**) Baseline sociodemographic, anthropometric, and hemodialysis characteristics of patients and distribution of study variables by sex. (**B**) Functional, morphological, and nutritional assessment. Comparison of functional performance, muscle ultrasound, bioimpedance, biochemical, and nutritional assessment parameters between women and men.

(**A**)				
	**Women** ***n* = 22** **29.7%**	**Men** ***n* = 52** **70.3%**	**Total** ***n* = 74** **100%**	***p*** **-Value**
Sociodemographic and anthropometric				
Age (years), mean (SD)	69.5 (19.8)	74.7 (13.3)	73.1 (15.5)	0.2
BMI (kg/m^2^), mean (SD)	26.1 (5.1)	24.4 (3.7)	24.9 (4.2)	0.11
Arm circumference (cm), mean (SD)	28.9 (3.4)	28.7 (8.6)	28.8 (7.4)	0.91
Calf circumference (cm), mean (SD)	81.8 (12.9)	76.1 (15.3)	77.8 (14.8)	0.13
Triceps skinfold (mL), mean (SD)	15.1 (5.8)	11.2 (6.0)	12.4 (6.2)	0.01
Suprailiac skinfold (mL), mean (SD)	17.9 (8.1)	16.3 (7.9)	16.7 (8.0)	0.42
COPD, *n* (%)	5 (22.7)	18 (34.6)	23 (31.1)	0.3
Ischemic heart disease, *n* (%)	9 (40.9)	28 (53.9)	37 (50.0)	0.3
Secondary hyperparathyroidism, *n* (%)	21 (95.5)	52 (100)	73 (98.7)	0.1
Causes of CKD, *n* (%)				
Diabetic kidney disease	7 (31.8)	18 (34.6)	25 (33.8)	0.8
Non-diabetic kidney disease	15 (68.2)	34 (65.4)	49 (66.2)	
Hemodialysis parameters				
HD vintage (months), mean (SD)	51.2 (41.7)	33.5 (27.2)	38.8 (32.9)	0.03
Dry weight (Kg), mean (SD)	63.8 (13.2)	70.1 (11.6)	68.2 (12.3)	0.047
IDWG (kg)-mean (SD	2.0 (0.6)	2.1(0.7)	2.1(0.7)	0.39
Kt/V urea, mean (SD)	1.7 (0.3)	1.6 (0.2)	1.6 (0.2)	0.02
KT (L)-mean (SD)	52.2 (5.9)	52 (6.6)	52 (6.4)	0.88
nPCR (g Urea/Kg/d), mean (SD)	1.1 (0.4)	1.1 (0.4)	1.1 (0.4)	0.63
QB (mL/min), mean (SD)	334.4 (22.6)	335.4 (21.1)	335.1 (21.4)	0.86
Inf.Vol. OL-HDF (L), mean (SD)	24.9 (4.1)	24.2 (4.5)	24.4 (4.4)	0.53
APF (mL/min), mean (SD)	182.1 (32.2)	168.9 (37.4)	172.8 (36.2)	0.16
VPF (mL/min), mean (SD)	167.4 (22.6)	160.2 (22.4)	162.3 (22.5)	0.21
SBP (mmHg), mean (SD)	136.1 (21.5)	128 (24.4)	130.4 (23.7)	0.18
DBP (mmHg), mean (SD)	69.9 (14.9)	66.5 (16.9)	67.5 (16.3)	0.41
Sessions per week (day)				
Three times per week, *n* (%)	18 (81.8)	43 (82.7)	61 (82.4)	0.9
iHD one or two times per week, *n* (%)	4 (18.2)	9 (17.3)	13 (17.6)	
Vascular access type				
Arteriovenous fistula, *n* (%)	6 (27.3)	25 (48.1)	31 (41.9)	0.1
Tunneled catheter, *n* (%)	16 (72.7)	27 (51.9)	43 (58.1)	
(**B**)				
	**Women** ***n* = 22** **29.7%**	**Men** ***n* = 52** **70.3%**	**Total** ***n* = 74** **100%**	***p*** **-Value**
Muscle strength				
Handgrip strength (kg), mean (SD)	11.9 (6.7)	20.9 (8.1)	18.2 (8.7)	<0.001
30 s Chair Stand Test (number of repeats), mean (SD)	9 (5.5)	10.1 (4.8)	9.8 (5)	0.41
Functional performance				
SPPB (points)	8.3 (2.2)	8.9 (2.2)	8.7 (2.2)	0.3
Low performance (SPPB ≤ 8), *n* (%)	6 (26.9)	15 (28.84)	21 (28.4)	0.7
Muscle nutritional ultrasound				
*Y*-axis (mm), mean (SD)	8.4 (2.2)	9.2 (2.4)	8.7 (2.3)	0.18
*Y*-axis/height (mm/m^2^), mean (SD)	3 (0.9)	3.8 (0.9)	3.2 (1)	<0.001
*Y*-axis/BSA (mm/m^2^), mean (SD)	2.7 (0.8)	3.5 (0.7)	2.9 (0.9)	<0.001
*X*-axis (mm), mean (SD)	30.5 (5.8)	30.7 (8.1)	30.5 (6.5)	0.89
CS-MARF (cm^2^), mean (SD)	2.5 (0.7)	2.9 (0.9)	2.6 (0.8)	0.03
MARFI_h_ (cm^2^/m^2^), mean (SD)	0.9 (0.3)	1.2 (0.4)	1 (0.3)	<0.001
MARFI_BSA_ (cm^2^/m^2^), mean (SD)	0.8 (0.2)	1.1 (0.3)	0.9 (0.3)	<0.001
*X*-axis/*Y*-axis ratio, mean (SD)	3.6 (1.5)	3.4 (0.9)	3.8 (1.4)	0.14
SMF (mm), mean (SD)	8.3 (2.5)	6.6 (2.0)	6.9 (2.2)	0.01
Visceral fat nutritional ultrasound				
Transverse PPVF, (cm) (SD)	0.6 (0.3)	0.6 (0.3)	0.6 (0.3)	0.71
Transverse SSCF (cm), mean (SD)	1.2 (0.5)	1.0 (0.4)	1.2 (0.5)	0.1
Transverse DSCF (cm), mean (SD)	0.9 (0.3)	0.8 (0.3)	0.8 (0.3)	0.3
Bioimpedance parameters				
TBW (L), mean (SD)	29 (3.9)	37.7 (7.9)	35.1 (8)	<0.001
ICW (L), mean (SD)	17.5 (2.6)	24.1 (9)	22.1 (8.2)	0.001
ECW (L), mean (SD)	11.4 (1.5)	15.3 (3.9)	14.1 (3.8)	<0.001
ECW/TBW ratio, mean (SD)	0.4 (0)	0.4 (0.1)	0.4 (0)	0.36
BFM (kg), mean (SD)	24.1 (11.4)	19.3 (8.5)	20.8 (9.6)	0.05
FFM (kg), mean (SD)	39.5 (5.4)	51.1 (11.2)	47.7 (11.2)	<0.001
LBM (kg), mean (SD)	36.6 (5.4)	48.2 (10.2)	44.7 (10.5)	<0.001
BCM (kg), mean (SD)	30.5 (7.0)	30.6 (6.5)	30.5(6.9)	0.97
BTM (kCals/24 h), mean (SD)	1222.7 (116)	1486.7 (218.6)	1408.2 (228.1)	<0.001
Skeletal muscle mass (kg), mean (SD)	20.9 (3.4)	28 (5.8)	25.9 (6.1)	<0.001
Bone mineral content (kg), mean (SD)	2.4 (0.3)	3.1 (0.6)	2.9 (0.6)	<0.001
TBW/FFM (%), mean (SD)	73.6 (0.5)	73.6 (0.9)	73.6 (0.8)	0.82
Protein (Kg), mean (SD)	7.6 (1.1)	9.9 (1.9)	9.2 (2)	<0.001
Minerals (Kg) mean (SD)	2.8 (0.4)	3.7 (0.7)	3.4 (0.7)	<0.001
Visceral fat area (cm^2^), mean (SD)	122.8 (67)	80.1 (46.3)	92.8 (56.3)	0.002
ASMI using Lin’s formula, kg/m^2^, mean (SD)	5.9 (0.8)	6.8 (1.2)	6.6 (1.2)	0.003
Phase angle (◦), mean (SD)	4.6 (1.4)	4.9 (1.5)	4.8 (1.5)	0.38
Biochemical parameters				
Hb (g/L), mean (SD)	10.7 (1.4)	11.4 (1.6)	11.2 (1.6)	0.07
Lymphocytes (10^3^/µL), mean (SD)	1.3 (0.4)	1 (0.5)	1.1 (0.5)	0.08
Fe (mg/dL), mean (SD)	70.3 (42.5)	69.7 (29)	69.8 (33.3)	0.94
Transferrin (mg/dL), mean (SD)	169.5 (28.9)	169.3 (29.8)	169.4 (29.4)	0.97
TSAT (%),	32.5 (18.6)	32.6 (13.6)	32.6 (15.1)	0.98
Ferritin (ng/mL), mean (SD)	541.5 [383–688]	679.5 [447–1101.5]	638.5 [413–1017]	0.28
Calcium serum (mg/dL), mean (SD	8.9 (0.8)	8.8 (0.7)	8.9 (0.7)	0.62
Magnesium serum (mg/dL), mean (SD)	2.2 (0.3)	2.2 (0.3)	2.2 (0.3)	0.75
Phosphorus serum (mg/dL), mean (SD)	4.2 (1.7)	4.3 (1.5)	4.3 (1.5)	0.69
25OHD serum (ng/mL), mean (SD)	26.5 (14.4)	25.1 (13.8)	25.5 (13.9)	0.7
PTH serum (pg/mL), mean (SD)	137 [73.7–415]	241 [109.5–340.8]	217.5 [86.6–346]	0.84
Cholesterol (mg/dL), mean (SD)	150.5 (38.2)	121.2 (24)	129.9 (31.7)	<0.001
Triglycerides (mg/dL), mean (SD)	130 (50.3)	119.8 (74.9)	122.8 (68.3)	0.56
HDL (mg/dL), mean (SD)	50.2 (21.9)	47.5 (16.3)	48.3 (18)	0.56
Non-HDL cholesterol (mg/dL), mean (SD)	100.3 (32.7)	73.6 (20.7)	81.6 (27.5)	<0.001
LDL (mg/dL), mean (SD)	80.6 (29.3)	53.7 (18.2)	61.7 (25.2)	<0.001
C-reactive protein (mg/L), mean (SD)	2.9 (7.4)	2 (3)	2.3 (4.8)	0.42
Albumin (g/dL), mean (SD)	3.3 (0.5)	3.2 (0.5)	3.2 (0.5)	0.77
Prealbumin (mg/dL), mean (SD)	26.5 (6.3)	26.5 (6.9)	26.5 (6.7)	0.99
Total proteins (g/dL), mean (SD)	6.4 (0.4)	6.4 (0.7)	6.4 (0.6)	0.8
Urea serum (mmol/mL), mean (SD)	111.9 (48.4)	122.3 (54.4)	119.2 (52.6)	0.44
Cr serum (g/dL), mean (SD)	5.7 (2.5)	6.2 (2.1)	6 (2.2)	0.42
Sodium serum (mmol/L), mean (SD)	138.1 (3.2)	138.2 (3.5)	138.2 (3.4)	0.97
Potassium serum (mmol/L), mean (SD)	4.6 (0.9)	4.5 (0.7)	4.5 (0.8)	0.92
Chlorine serum (mmol/L), mean (SD)	101.9 (3.1)	101.6 (4)	101.7 (3.7)	0.73
Bicarbonate (mEq/L), mean (SD)	22.5 (2.6)	23.6 (2.3)	23.3 (2.4)	0.09
Scales of risk malnutrition				
7 –points SGA scale				
Well-nourished, *n* (%)	13 (59)	24 (46)	37 (50)	0.06
Mild–moderate–severely malnourished, *n* (%)	9 (41)	28 (54)	37 (50)	
MIS (points)	7.9 (3.3)	7.5 (4.1)	7.6 (3.9)	0.7
Patients with MIS ≥ 8 points, *n* (%)	9 (40.9)	21 (40.4)	30 (40.5)	0.97
MST ≥ 2 points, *n* (%)	3 (13.6)	13 (25)	16 (21.6)	0.3
PEW (score)				
PEW (score 0–3), *n* (%)	19 (86.4)	47 (90.4)	66 (89.2)	0.6
No PEW (score 4), *n* (%)	3 (13.6)	5 (9.61)	8 (10.8)	
FRAIL scale				
No frailty (score 0 points), *n* (%)	7 (31.8)	23 (44.2)	30 (40.5)	0.5
Risk of frailty (score 1–2 points), *n* (%)	6 (27.3)	9 (17.3)	15 (20.3)	
Frail (score ≥ 3 points), *n* (%)	9 (40.9)	20 (38.5)	29 (39.2)	
SARC-F score	3.4 (2.9)	2.6 (2.9)	2.9 (2.9)	0.28
SARC-F ≥ 4 points, *n* (%)	9 (40.9)	17 (32.7)	26 (35.1)	0.5
EWGSOP2				
Confirmed sarcopenia, *n* (%)	5 (22.7)	25 (48.1)	30 (40.5)	0.01
Risk of sarcopenia, *n* (%)	10 (45.5)	8 (15.4)	18 (24.3)	
Non-sarcopenia, *n* (%)	7 (31.8)	19 (36.5)	26 (35.1)	

Data are shown as mean (SD: standard deviation) or median [interquartile range—IQR] or *n* = number/percentage (%). BMI: body mass index; COPD: chronic obstructive pulmonary disease; CKD: chronic kidney disease; HD: hemodialysis; IDWG: interdialytic weight gain; QB: blood flow rate; nPCR: normalized protein catabolic rate; Inf.Vol. OL-HDF: infusion volume in post-dilution online hemodiafiltration; APF: arterial pressure flow; VPF: venous pressure flow; SBP: systolic blood pressure; DBP: diastolic blood pressure; iHD: incremental hemodialysis. SSPB: short physical performance battery; BSA: body surface area; CS-MARF: cross-sectional muscle area of the rectus femoris; MARFIh: muscle area of the rectus femoris index adjusted to height; MARFIBSA: muscle area of the rectus femoris index adjusted to body surface area; SMF: supramuscular fat; PPVF: preperitoneal visceral fat; SSCF: superficial subcutaneous fat; DSCF: deep subcutaneous fat; TBW: total body water; ICW: intracellular water; ECW: extracellular water; BFM: body fat mass; FFM: fat-free mass; LBM: lean body mass; BMR: basal metabolic rate; BCM: body cell mass; ASMI: appendicular skeletal mass index; Hb: Hemoglobin; Fe: iron; TSAT: transferrin saturation; Cr: creatinine; 25OHD: 25-hidroxy vitamin D; PTH: parathyroid hormone; HDL: high-density lipoprotein; LDL: low-density lipoprotein; SGA: subjective global assessment; MIS: malnutrition–inflammation score; MST: malnutrition screening tool; PEW: protein–energy wasting; FRAIL: fatigue, resistence, ambulation, illnesses, and loss; SARC-F: strength, assistance with walking, rising from a chair, climbing stairs, and falls; EWGSOP2: European Working Group on Sarcopenia in Older People 2.

**Table 2:** (**A**) Comparison of demographic, functional, biochemical, and clinical variables among patients with non-sarcopenia, risk of sarcopenia, and confirmed sarcopenia. (**B**) Comparison of nutritional ultrasound and bioimpedance-derived morphological variables among patients stratified by sarcopenia status.

(**A**)				
**N (%)**	**Non-Sarcopenia** **26 (35.1)**	**Risk-Sarcopenia** **18 (24.3)**	**Confirmed-Sarcopenia** **30 (40.5)**	***p*** **-Value**
Anthropometry variables				
Age—years, mean (SD)	64.8 (16.9)	71.3 (9.7)	81.5 (15.5)	<0.001
Sex—male, (%)	73.1	44.4	83.3	0.02
Weight (kg), mean (SD)	73.3 (10.4)	72.4 (10.1)	61.3 (12.3)	<0.001
BMI (kg/m^2^), mean (SD)	26.9 (4.1)	28.1 (4.1)	22.1 (2.5)	<0.001
Triceps skinfold (mL), mean (SD)	13.1 (6.7)	15.4 (4.8)	9.9 (6.2)	0.006
Suprailiac skinfold (mL), mean (SD)	19.7 (8.5)	19.5 (4.9)	12.5 (8)	<0.001
Muscle strength				
Handgrip strength (kg), mean (SD)	26.8 (6.8)	12 (6.1)	14.5 (8.7)	<0.001
Functional performance				
SPPB (points)	10.3 (1.5)	8.2 (2)	7.6 (2)	<0.001
Low performance (SPPB ≤ 8), (%)	3.9	27.8	50	0.001
Hemodialysis parameters				
HD vintage (months), mean (SD)	33 (28.5)	39 (37.2)	43.6 (32.9)	0.49
nPCR (g Urea/Kg/d), mean (SD)	1.3 (0.2)	0.9 (0.4)	1 (0.4)	0.001
Vascular access type				
Arteriovenous fistula, (%)	46.2	44.4	36.7	0.75
Tunneled catheter, (%)	53.9	55.6	63.3	
Kt/V urea, mean (SD)	1.6 (0.2)	1.6 (0.2)	1.6 (0.2)	0.95
KT (L), mean (SD)	54 (5.8)	53.2 (5.3)	49.7 (6.4)	0.02
HD conventional (three sessions/week), *n* (%)	20 (76.9)	16 (88.9)	25 (83.3)	0.6
iHD (one or two sessions/week), *n* (%)	6 (23.1)	2 (11.1)	5 (16.7)	
Biochemical parameters				
Hb (g/L), mean (SD)	11 (1.6)	10.7 (1.5)	11.7 (1.6)	0.06
Lymphocytes (10^3^/µL), mean (SD)	1.1 (0.5)	1.2 (0.5)	1.1 (0.5)	0.6
Ferritin (ng/mL), mean (SD)	657.6 (386.3)	841.7 (709.7)	848.6 (520.1)	0.3
sCr (g/dL), mean (SD)	7.1 (2.3)	5.8 (2)	5.3 (2.2)	0.007
Cholesterol (mg/dL), mean (SD)	122.3 (37.3)	138.2 (30.7)	131.5 (31.7)	0.25
Triglycerides (mg/dL), mean (SD)	133.7 (91.4)	151.6 (34.4)	96.2 (68.3)	0.01
C-reactive protein (mg/L), mean (SD)	2.2 (3.8)	2.3 (3.4)	2.3 (4.8)	0.99
Albumin (g/dL), mean (SD)	3.4 (0.5)	3.1 (0.5)	3.2 (0.5)	0.13
Prealbumin (mg/dL), mean (SD)	27.7 (5.8)	26.1 (5.4)	25.6 (6.7)	0.51
(**B**)				
**N (%)**	**Non-Sarcopenia** **26 (35.1)**	**Risk-Sarcopenia** **18 (24.3)**	**Confirmed-Sarcopenia** **30 (40.5)**	***p*** **-Value**
Bioimpedance parameters				
TBW (L), mean (SD)	39.4 (9.5)	34.7 (5.7)	31.7 (8)	0.001
BFM (kg), mean (SD)	19.7 (10)	26.8 (6.9)	18.1 (9.6)	0.007
FFM (kg), mean (SD)	53.5 (12.4)	47 (9.2)	43 (11.2)	0.002
LBM (kg), mean (SD)	49.4 (12.4)	43.7 (7.3)	41.3 (10.5)	0.01
BCM (kg), mean (SD)	34.3 (7.4)	29.8 (5.5)	27.6 (6.9)	0.001
Skeletal muscle mass (kg), mean (SD)	29.1 (6.7)	25.2 (4.8)	23.4 (6.1)	0.001
Visceral fat area (cm^2^), mean (SD)	85.3 (63.4)	124.1 (43.6)	80.4 (56.3)	0.02
ASMI using Lin’s formula (kg/m^2^), mean (SD)	7.2 (1.2)	7 (0.7)	5.8 (1.2)	<0.001
ASMI < 5.5 kg/m^2^ (♀) and < 7 kg/m^2^ (♂), *n* (%)	1 (3.8)	7 (38.9)	18 (60)	<0.001
Phase angle (◦), mean (SD)	5.2 (2)	5.1 (0.8)	4.3 (1.5)	0.03
Nutritional ultrasonography parameters				
*Y*-axis (mm), mean (SD)	9.6 (2.8)	8.8 (2)	7.8 (2.3)	0.01
*Y*-axis/height (mm/m^2^), mean (SD)	3.4 (1.3)	3.5 (0.7)	2.9 (1)	0.03
*Y*-axis/BSA (mm/m^2^), mean (SD)	2.9 (1.1)	3 (0.8)	2.9 (0.9)	0.91
*X*-axis (mm), mean (SD)	31.6 (6)	29.5 (6.5)	30.2 (6.5)	0.54
*X*-axis/*Y*-axis ratio, mean (SD)	1.2 (0.3)	1.4 (0.7)	1.5 (0.5)	0.09
CS-MARF (cm^2^), mean (SD)	2.9 (1)	2.6 (0.6)	2.4 (0.8)	0.03
MARFI_h_ (cm^2^/m^2^), mean (SD)	1 (0.4)	1 (0.3)	0.9 (0.3)	0.13
MARFI_BSA_ (cm^2^/m^2^), mean (SD)	0.9 (0.3)	0.9 (0.3)	0.9 (0.3)	0.88
SMF (mm), mean (SD)	7.5 (2.1)	7.3 (1.8)	6 (2.2)	0.02
Transverse PPVF (cm), mean (SD)	0.7 (0.3)	0.6 (0.2)	0.5 (0.3)	0.05
Transverse SSCF (cm), mean (SD)	0.9 (0.3)	0.8 (0.2)	0.7 (0.3)	0.04
Transverse DSCF (cm), mean (SD)	1.1 (0.6)	1.4 (0.4)	1.1 (0.5)	0.05
Scales of malnutrition, frailty, and sarcopenia				
7-point SGA (malnutrition), *n* (%)	3 (11.5)	12 (66.7)	22 (73.3)	<0.001
MIS ≥ 8 points, *n* (%)	3 (11.5)	9 (50)	18 (60)	0.009
MST ≥ 2 points, *n* (%)	2 (7.7)	4 (22.2)	10 (33.3)	0.07
Frailty (score ≥ 3 points), *n* (%)	3 (11.5)	8 (44.4)	18 (60)	<0.001
Severe PEW (score 0–2), *n* (%)	6 (23.1)	7 (38.9)	20 (66.7)	0.004
SARC-F ≥ 4 points, *n* (%)	3 (11.5)	7 (38.9)	16 (53.3)	0.04

Data are shown as mean (SD: standard deviation) or median [interquartile range—IQR] or *n* = number/percentage (%). BMI: body mass index; SSPB: short physical performance battery; HD: hemodialysis; nPCR: normalized protein catabolic rate; iHD: incremental hemodialysis; Hb: hemoglobin; sCr: serum creatinine. TBW: total body water; BFM: body fat mass; FFM: fat-free mass; LBM: lean body mass; BCM: body cell mass; ASMMI: appendicular skeletal muscle mass index; BSA: body surface area; CS-MARF: cross-sectional muscle area of the rectus femoris; MARFIh: muscle area of the rectus femoris index adjusted to height; MARFIBSA: muscle area of the rectus femoris index adjusted to body surface area; SMF: supramuscular fat; PPVF: preperitoneal visceral fat; SSCF: superficial subcutaneous fat; DSCF: deep subcutaneous fat; SGA: subjective global assessment; MIS: malnutrition–inflammation score; MST: malnutrition screening tool; PEW: protein–energy wasting; FRAIL: fatigue, resistence, ambulation, illnesses, and loss; SARC-F: strength, assistance with walking, rising from a chair, climbing stairs, and falls.

**Table 3:** ROC analysis for US measures for the determination of sarcopenia.

	AUC	95% CI	Sign	Sensitivity (%)	Specificity (%)
*Y*-axis (mm)	0.67	0.54–0.79	*p* < 0.05	76.7	56.8
*Y*-axis/height (mm/m^2^)	0.65	0.52–0.77	*p* < 0.05	60	45.5
*Y*-axis/BSA (mm/m^2^)	0.51	0.37–0.65	NS	73.3	50
MARFI_h_ (cm^2^/m^2^)	0.63	0.50–0.75	*p* < 0.05	86.7	40.9
MARFI_BSA_ (cm^2^/m^2^)	0.50	0.36–0.63	NS	70	34.1
SMF (mm)	0.67	0.54–0.79	*p* < 0.05	96.7	31.8
Transverse PPVF (cm)	0.63	0.49–0.75	NS	53.3	65.9
Transverse SSCF (cm)	0.66	0.53–0.79	*p* < 0.05	53.3	70.5
Transverse DSCF (cm)	0.60	0.47–0.73	NS	60	56.8

AUC: area under the curve; CI: confidence interval; BSA: body surface area; MARFI_h:_ muscle area of the rectus femoris index adjusted to height; MARFI_BSA_: muscle area of the rectus femoris index adjusted to body surface area; SMF: supramuscular fat; PPVF: preperitoneal visceral fat; SSCF: superficial subcutaneous fat; DSCF: deep subcutaneous fat; NS: not significant.

## Data Availability

No new data were created or analyzed in this study. The data used to support the findings of this study are available from the corresponding author upon request (contact J.C.D.L.F., jflomer@mde.es). We confirm that all figures and tables are the original work of this manuscript’s authors. The authors created all content, which has not been adapted from other sources, and no online link is provided.
